# Rapid Mutation of *Spirulina platensis* by a New Mutagenesis System of Atmospheric and Room Temperature Plasmas (ARTP) and Generation of a Mutant Library with Diverse Phenotypes

**DOI:** 10.1371/journal.pone.0077046

**Published:** 2013-10-11

**Authors:** Mingyue Fang, Lihua Jin, Chong Zhang, Yinyee Tan, Peixia Jiang, Nan Ge, Heping Li, Xinhui Xing

**Affiliations:** 1 Key Laboratory for Industrial Biocatalysis, Ministry of Education, Department of Chemical Engineering, Tsinghua University, Beijing, China; 2 Department of Engineering Physics, Tsinghua University, Beijing, China; 3 Colloge of bioengineering, Beijing Polytechnic, Beijing, China; Louisiana State University, United States of America

## Abstract

In this paper, we aimed to improve the carbohydrate productivity of *Spirulina platensis* by generating mutants with increased carbohydrate content and growth rate. ARTP was used as a new mutagenesis tool to generate a mutant library of *S. platensis* with diverse phenotypes. Protocol for rapid mutation of *S. platensis* by 60 s treatment with helium driven ARTP and high throughput screening method of the mutants using the 96-well microplate and microplate reader was established. A mutant library of 62 mutants was then constructed and ideal mutants were selected out. The characteristics of the mutants after the mutagenesis inclined to be stable after around 9^th^ subculture, where the total mutation frequency and positive mutation frequency in terms of specific growth rate reached 45% and 25%, respectively. The mutants in mutant library showed diverse phenotypes in terms of cell growth rate, carbohydrate content and flocculation intensity. The positive mutation frequency in terms of cellular carbohydrate content with the increase by more than 20% percent than the wild strain was 32.3%. Compared with the wild strain, the representative mutants 3-A10 and 3-B2 showed 40.3% and 78.0% increase in carbohydrate content, respectively, while the mutant 4-B3 showed 10.5% increase in specific growth rate. The carbohydrate contents of the representative mutants were stable during different subcultures, indicating high genetic stability. ARTP was demonstrated to be an effective and non-GMO mutagenesis tool to generate the mutant library for multicellular microalgae.

## Introduction

Microalgae have been paid more attention because of their wide range of applications by diverse functions, such as in food, cosmetics and pharmaceutical industry [1-4]. Microalgae also can be used to treat wastewater to form biomass, thereby increasing the efficiency of microalgae productivity [1,5-9]. In recent years, microalgae as cell factories for production of biofuels and biochemicals have attracted increasing attention, due to their fixation of CO_2_ for reduction of the green-house gas[10]. As for biofuel production, there are two potential approaches for microalgae biotechnology: 1) harvesting lipid from the microalgae cells to produce bioiesel; 2) biofuel fermentation from the microalgae biomass capable of storing carbohydrate such as starch and glycogens, which can be used as non-edible feedstock for fermentation. Many studies have been carried out on cultivation of microalgae with the lipid content ranging from 20% to 50% to produce biodiesel[11]. Although microalgae have features such as relatively high growth rate, further efforts are required to screen or breed microalgae species with higher lipid content, efficiently harvest biomass, especially for the unicellular microalgae, establish the energy-saving lipid extraction process and develop the integrated system for economic biodiesel production [12-14]. On the other hand, using the carbohydrate-containing microalgae as the substrates for fermentation is also worth paying attention, since the saccharification of the whole microalgae biomass containing carbohydrate is relatively easily reachable for the subsequent fermentation[15-17]. For all these applications of microalgae, effective strain improvement technique is indispensable, especially non-GM (genetic modification) method for open-pond cultivation of microalgae. 

*Spirulina platensis* is a kind of filamentous cyanobacteria with multicellular cylindrical trichomes. This microalgae has many properties such as high protein content, unique composition of fatty acids and vitamins, and high carbohydrate content [18], indicating that *S. platensis* can be a suitable feedstock for fermentation. Also, *S. platensis* can be flocculated because of its filamentous form, which makes its harvesting easier than other microalgae[19]. The carbohydrate generally make up 15-20% of the dry weight of *S. platensis*, which are mainly branched sugars consisting of only glucose and structurally similar glycogen [20,21]. It has been revealed that glucose content in *S. platensis* reach 7-8% of dry cell weight [2], which will make the hydrolysis process much easier. In the last decade, *S. platensis* has been commercially produced for food supplement[22]. The mature outdoor culture method will provide a platform for *S. platensis* application as the fermentation feedstock. 

In order to make the biomass of *S. platensis* useful as fermentation feedstock, strain improvement for increasing the growth rate and carbohydrate content is still needed. The genetic engineering method is widely used to improve microbial performance, however it still has difficulties in modification of *S. platensis*[19], presumably due to the gliding motility on agar plate, lacking of the genetic modification tools and difficulty in introduction of the foreign DNA molecules[19,23,24]. Construction of a gene transfer system for *S. platensis* has been tried. Toyomizu et al. increased the transformation efficiency of electroporation by the optimized electric-field strength and time constant[25]. Kawata et al. applied a modified transformation strategy using a natural Tn5 transposon, transposase, and cation liposome complex by electroporation to improve the transformation efficiency[26]. However, instability of the transformants and low efficiency of foreign gene expression have been the major obstacles, so little success has been reached for efficient genetic engineering of *S. platensis* to improve its phenotypes.

As a non-GM mutation method, random mutagenesis has been a useful tool and widely used for generating mutants of different microorganisms. Different mutation techniques including chemical and physical mutagens have been applied to *S. platensis*. Singh et al. reported that after chemical mutagen (nitrosoguanidine, NTG) treatment, the isolated mutant of *S. platensis* cells exhibited approximately three-fold higher tolerance to metronidazole and DCMU (3,4-dichlorophenyl-1, 1-dimethylurea) as compared with wild-type strain [27], but they didn’t improve the growth of *S. platensis*. With the same mutagen NTG, Riccardi et al. isolated *S. platensis* mutants resistant to 5-fluorotryptophan, β-2-thienyl-alanine, ethionine, p-fluprophenlalanine or azetidine-2-carboxylic acid. A few of the mutants overproduce the corresponding amino acids such as proline or valine[28,29], but the highest mutation frequency is 1.2~7.1×10^-6^ per plated filaments. Lanfaloni et al. tried to create the mutants of *S. platensis* resistant to 8-azaguanine or β-2-thienyl-DL-alanine treated by 1-3 min UV irradiation followed by incubation with 50 μg N-methyl-N-nitro-N-nitrosoguanidine (MNNG), but they could not isolate an ideal mutant [30]. What is more, all the chemical mutagenesis approaches have the safety problem to the operator and environment. Therefore, a new mutagenesis tool which is rapid, effective, safe and environmental benign is needed for effective mutation of *S. platensis*.

Atmospheric and room temperature plasma (ARTP) is a powerful and novel physical microbial mutagenesis tool for creating mutant library of microorganisms [31-33]. ARTP driven by the radio-frequency power can be generated uniformly at atmospheric pressure without any vacuum system and the plasma can be controlled at room temperature, which is beneficial for microbial mutation. Active chemical species with the high density are the main composition of ARTP, which can penetrate the cell wall and membrane, damage DNA molecules, cause the mutation and thereby alter the metabolic networks of the target microbes[32-36]. An automated ARTP mutation breeding system has been invented by our group [32], which is compact and can be operated safely and feasibly, enabling rapid and diverse genome mutation of microbes in a non-GMO manner.

In this paper, we aimed to mutate *S. platensis* to generate the mutants with high carbohydrate content and high growth rate by ARTP mutation system, which are indispensable for increasing the productivity of *S. platensis* biomass as the fermentation feedstock. We applied the ARTP mutagenesis system to establish the mutation and a high throughput screening procedure for *S. platensis* to generate the mutant library. Then, *S. platensis* mutants with different carbohydrate contents and growth rates were selected out and examined.

## Materials and Methods

### 1: Materials

*Spirulina platensis*, the wild type strain (*S. platensis* FACHB-904, Freshwater Algae Culture Collection of the Institute of Hydrobiology, Wuhan, China) and the mutants generated by ARTP, were routinely grown in Zarrouk medium [37] by static cultivation in an artificial climate incubator at 28 °C with white fluorescent lamp set light intensity of 49~57 μmol·m^-2^·s^-1^, with a cycle of 10 h light/14 h dark for the culture. 2. Mutation procedure of *S. platensis* by ARTP 

**Figure 1:**
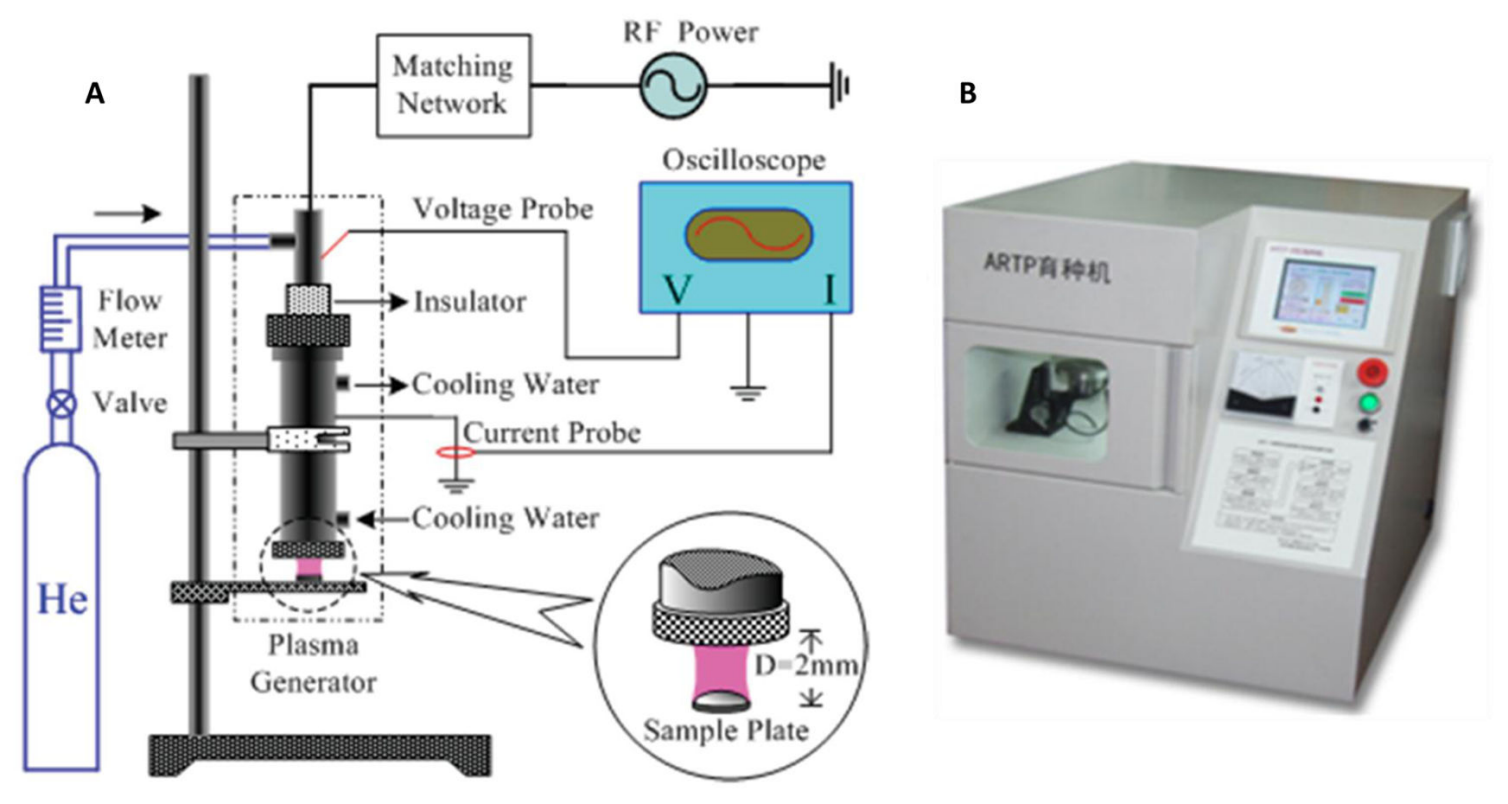
ARTP mutagenesis system. Schematic diagram of ARTP (A) and outlook of the instrument (B).

The ARTP mutation system (Figure 1) consisted of an RF(13-56 MHz) power supply subsystem, a co-axial type plasma generator, a gas supply control subsystem, and a simple plate made of stainless steel [33,35,36]. In this study, pure helium was used as the plasma working gas and the operating parameters were as follows: 1) The RF power input was 100 W, 2) the distance between the plasma torch nozzle exit and the sample plate (*D*) was 2 mm; 3) the temperature of the plasma jet was 25-35 °C. Under these determined conditions, the mutagenesis dosage by ARTP was dependent on the treatment period. To determine the optimal treatment period, *S. platensis* grown at OD_560_ of 0.8~1.0, which was within the log-phase, was selected to perform ARTP treatment. For the mutation, 10 μl of the culture was dipped onto the stainless steel minidisc and then exposed to ARTP jet for 5, 10, 20, 40, 60 and 80 s, respectively.

After the ARTP treatment for different time, the *S. platensis* samples were all washed by 10-20 μl fresh broth and moved to the inverted microscope to observe the change of morphology. The *S. platensis* filaments were cut into short fragments because of the action of the active plasma generated by ARTP on the cell walls of *S. platensis* and the fragments with green color were considered to be survived. The spirals of each green *S. platensis* fragments were counted and the total number of spirals was calculated. The survival rate was estimated by dividing the number of the total spirals survived after ARTP treatment by the number of the total spirals before ARTP treatment and then multiplied it by 100%. 

### 3: High throughput screening of the mutants

**Figure 2:**
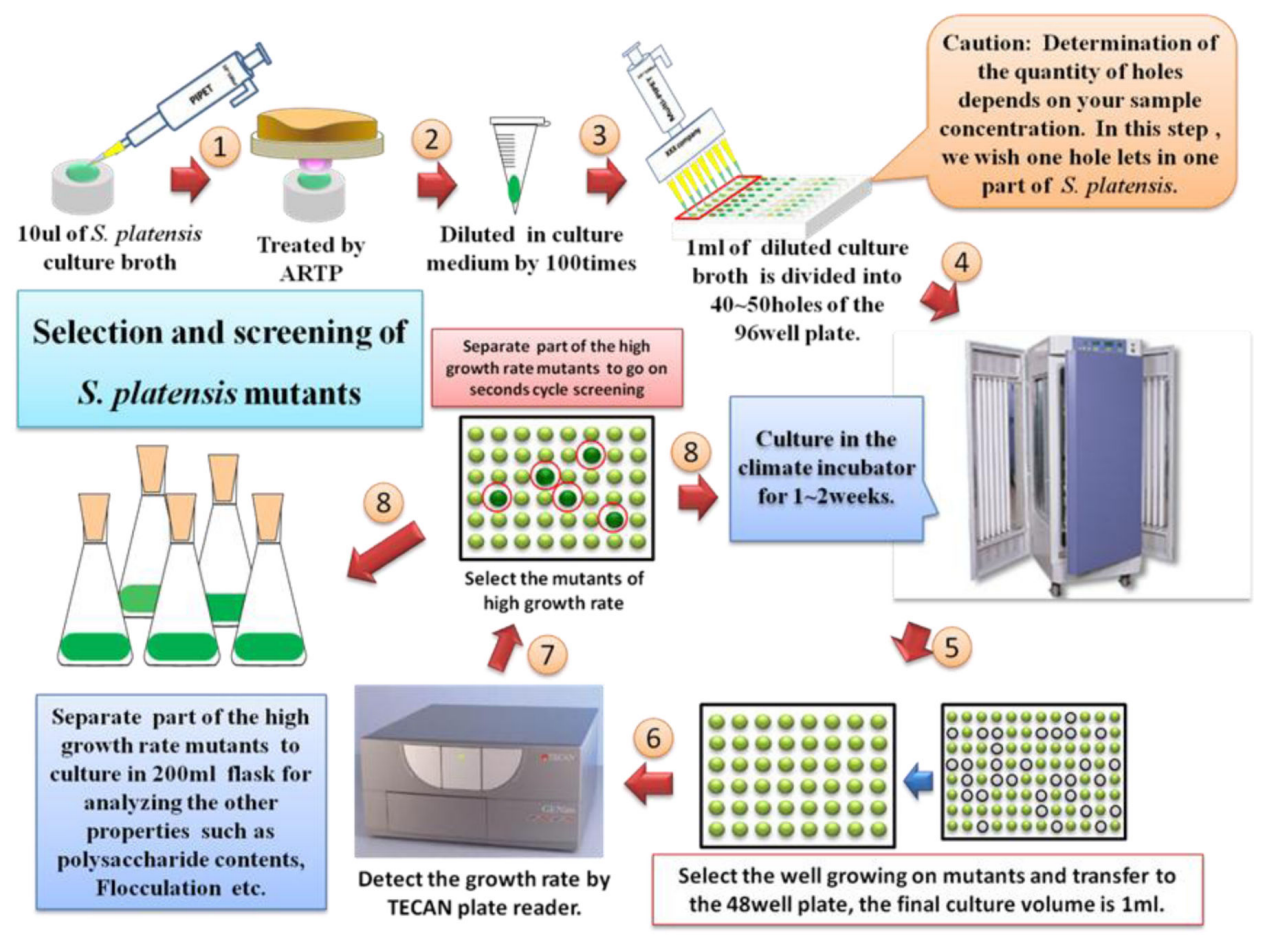
The experimental protocol for high throughput screening of the *S. platensis* mutants generated by ARTP.

Since *S. platensis* is a kind of multicellular microalgae, after ARTP treatment, the long spirals or filaments were cut into short ones, the efficient separation of the short filaments by controlling the smallest spiral number into a well of microplate will ensure the efficiency to rapidly screen the mutants by aiding the microplate reader. Figure 2 shows the established high throughput screening process: 1) after ARTP treatment, ARTP-treated *S. platensis* was first diluted with the fresh medium by different dilution rates; 2) the diluted *S. platensis* solution was transferred to each well of 96-well plates to make sure that each hole contained at most one filament. This process was confirmed by a microscope (Nikon, Japan); 3) after a period of cell division and growth, some wells exhibited green color while some holes showed blank without *S. platensis* growing. The blank one was abandoned and the green one was picked out and transferred to 48-well plates for further screening culture. During the screening process with the 48-well plates, we detected OD_560_ value of each well for generating the growth curve of mutants by a microplate reader (Tecan, Switzerland). The wild strain was also cultivated in the microplate wells for comparison. After several times of subculture with the 48-well microplates where one subculture was carried out for 10 d in the artificial climate incubator, when the characteristics of the mutants became stable, all the mutants and wild strain were transferred into 50 ml flasks containing 20 ml medium for further cultivation of 10 d to detect the carbohydrate content and flocculation intensity of each strain. After establishing the mutant library, the representative mutants were chosen and cultivated in 200 ml flasks each containing 50 ml medium of two weeks to analyze the specific growth rate, flocculation intensity and carbohydrate content precisely. The genetic stability of the selected mutants was also examined by a series of subcultures for the carbohydrate content detection.

### 4: Analysis

#### 4.1: Biomass specific growth rate and mutation frequency

The biomass concentration was determined by measuring the OD_560_ value of the culture at 560nm with a microplate reader (Tecan, Switzerland) using the 48-wells plates. Specific growth rate (*μ*) was calculated by following Eq. (1)[24].

$$\text{μ=}\frac{{\text{lnx}}_{\text{2}}{\text{-lnx}}_{\text{1}}}{t_{2}-t_{1}}$$ (1)

where, *x*_*1*_ and *x*_*2*_ were the biomass concentration in terms of dry cell weight at time intervals *t*_*1*_ and *t*_*2*_. Biomass concentration of dry cell weight was calculated by following Eq. (2)

y=1.251x-0.01925 (2)

where, x and y were biomass concentration (g/L) and OD_560_ value, respectively, which was obtained from the relationship between dry cell weight and OD_560_ value.

The mutant whose growth rate was higher or lower than that of the wild strain by 20% was defined as the an effective mutant, and the percentage of the effective mutants in the mutant library was defined as mutation frequency. The mutant whose growth rate was higher than that of the wild type by 20% was defined as a positive mutant, and the percentage of the positive mutants in the library was defined as positive mutation frequency. 

#### 4.2: Carbohydrate content

After biomass culture was collected by centrifugation at 13000×g for 10 min, 6 N HCl solution of same volume was used to break up the cell walls of *S. platensis* and hydrolyze the intracellular carbohydrate at 95°C for 10 min, then 6 N NaOH of the same volume were added to neutralize the solution. The formed reduced sugar was detected by the DNS methods for calculating the carbohydrate content [38].

#### 4.3: Chlorophyll assay

The chlorophyll(Chl) content was measured according to the method proposed by Lee [39] and Chen [40]. The 5 ml *S. platensis* spirals were collected by centrifugation at 13000×g for 10 min. The collected cells were washed 3 times by 0.9% NaCl solution, followed by the addition of 5 ml of 90% methanol solution, and then sonication. The extracted solution was measured at 665 and 650nm by UV-spectrophotometer, respectively, and the Chl content (mg/L) was evaluated according to Eq. (3) [39,40]

$$\text{Chl content=16}\text{.5}\times {\text{OD}}_{\text{665}}\text{-8}\text{.3}\times {\text{OD}}_{\text{650}}$$ (3)

#### 4.4: Flocculation intensity

For detection of the flocculation of *S. platensis*, optical density of the supernatant (A_S_) and mixture (A_M_) of the culture broth were detected at 560nm by UV-spectrophotometer (GE, New York, USA). The degree of flocculation intensity (F) was calculated by Eq. (4).

$$F=\frac{A_{M}-A_{S}}{A_{M}}\times 100\%$$ (4)

## Results

### 1: Mutation procedure setup and assessment

**Figure 3:**
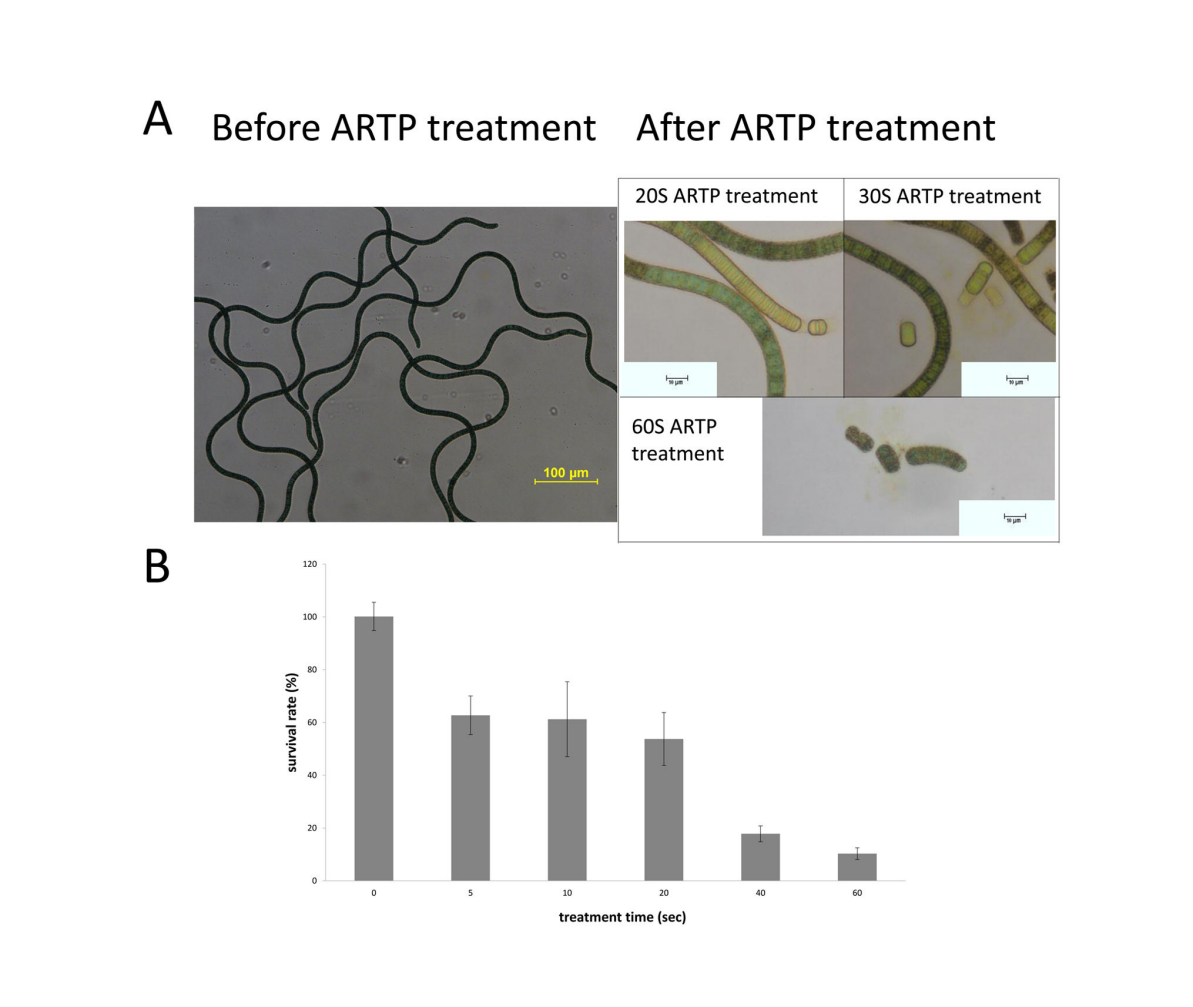
Change of *S. platensis* after treatment of ARTP. Change of morphology (A) and survival rate (B) of *S. platensis* during ARTP treatment (n=3).

**Figure 4:**
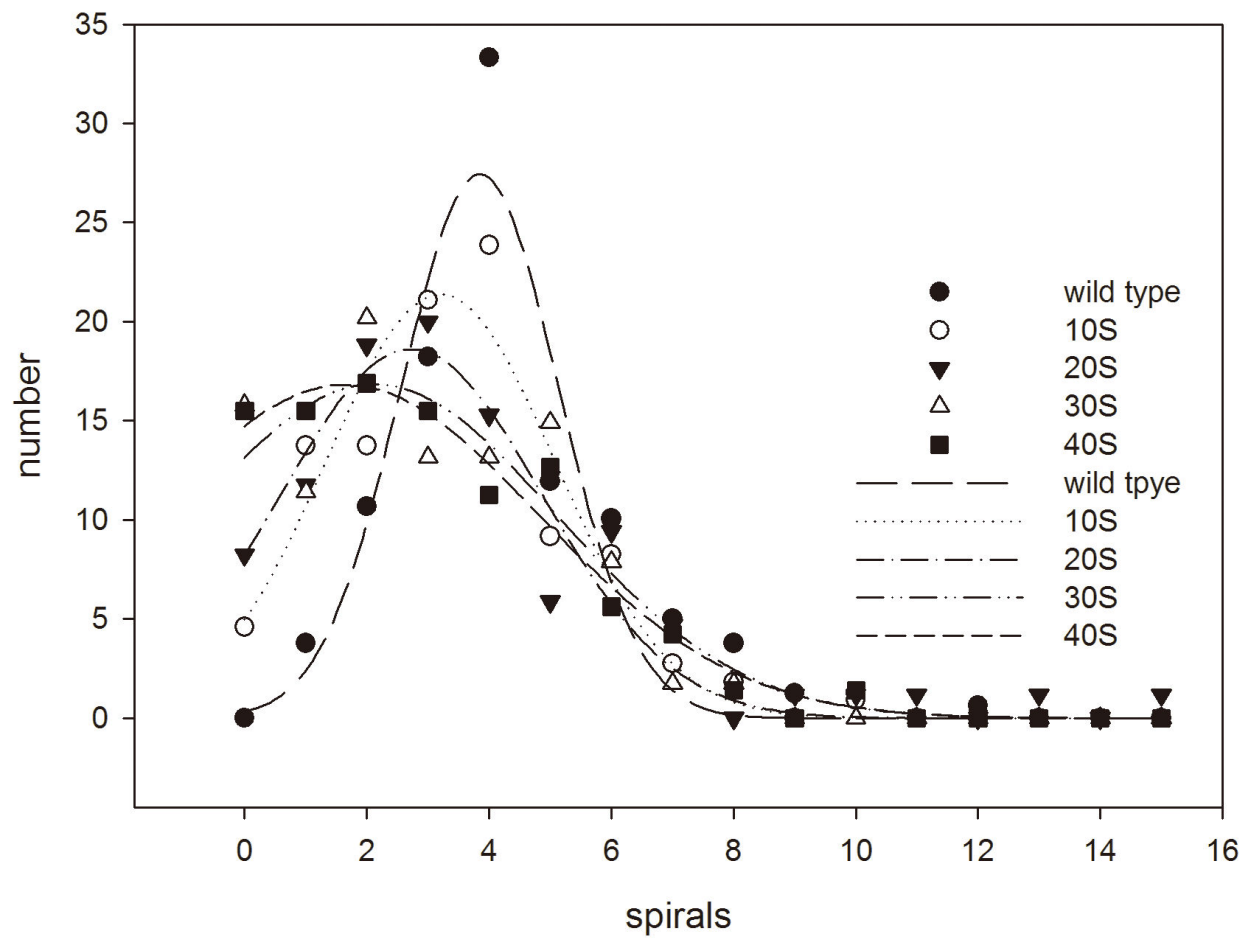
Change in the number distribution of *S. platensis* spirals with increase of ARTP mutagenesis dosage. In this figure, the length of *S. platensis* was calculated by spiral numbers in one filament. The number distribution of spirals during the ARTP treatment was regressed by Gaussian distribution, X_0_: peak, R: Regression, SEE：Standard Error of Estimate (Table 3).

In this study, *S. platensis* was mutated by the new mutation tool of ARTP (Figure 1). For generating a mutant library, the ARTP mutagenesis condition was firstly determined by detecting the survival rate and morphology change of *S. platensis*. The survival rate decreased to 10.28% when the treatment time was 60 s (Figure 3B). Meanwhile, morphology was also an important feature of *S. platensis*, as shown in Figure 3A, after the ARTP treatment, the long spirals of *S. platensis* were cut into short ones, the broken pieces of *S. platensis* were presented as healthy green color when the treatment time was short, while with the increase of the treatment time, the spirals became fewer. When the treatment time exceeded 60 seconds, *S. platensis* spirals were all broken into small debris. Figure 4 shows the change in spiral number distribution of *S. platensis* before and after ARTP treatment for different treatment time by regressing with Gaussian distribution. The peak of spiral numbers of wild *S. platensis* without ARTP treatment was about 3.9, while after 40 seconds of ARTP treatment, the peak for the spiral numbers decreased to 1.6. When the treatment time was longer, the peak value of the spirals became lower. This result demonstrated that the ARTP treatment could cause breakage of the filaments of *S. platensis.*

**Table 3:** Values in Gaussian distribution of Figure. 4.

Time	0	10S	20S	30S	40S
Xo	3.8502	3.2050	2.7308	2.1184	1.6447
R	0.9429	0.9661	0.9619	0.9521	0.9882
SEE	3.2454	2.2099	2.0365	2.3971	1.1435

### 2: Mutant screening and quality assessment of the mutant library

**Figure 5:**
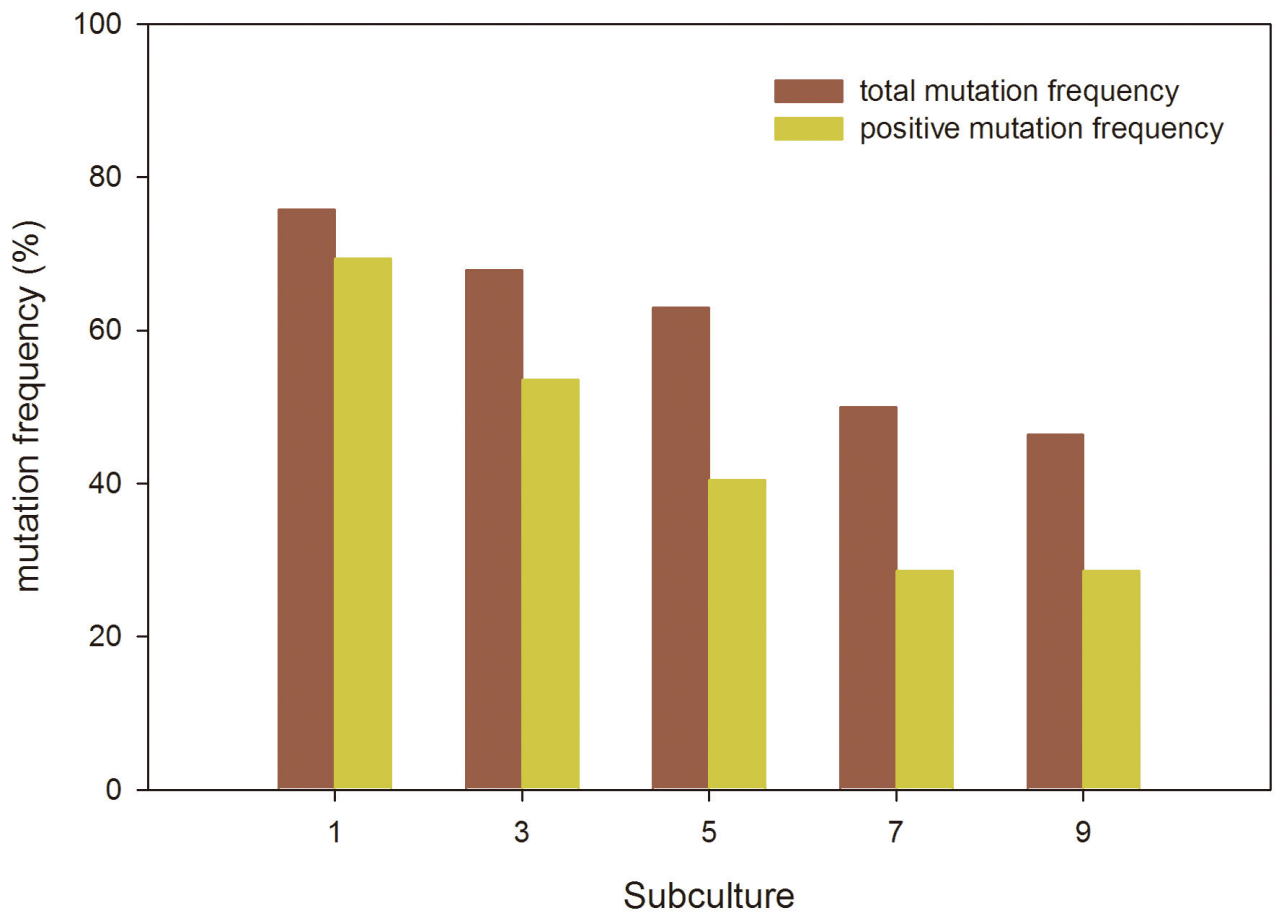
Change in mutation frequency in terms of specific growth rate during subcultures.

**Figure 6:**
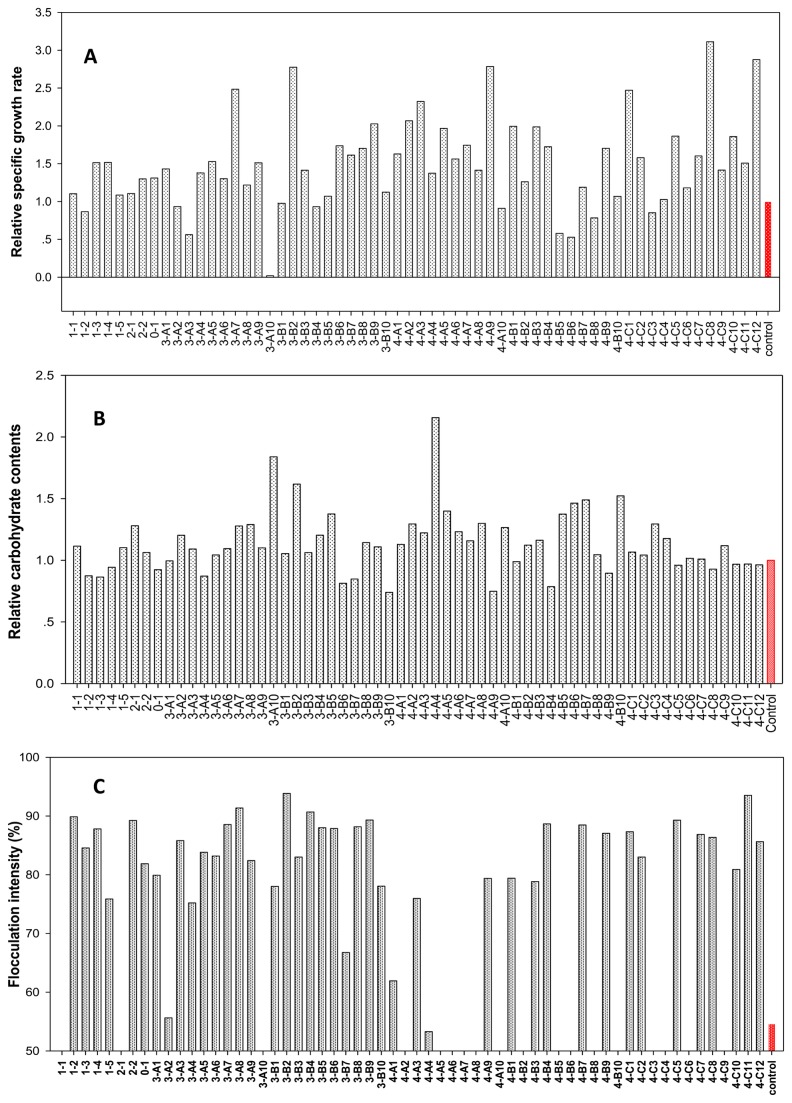
Characteristics of mutants in the mutant library. Comparison of the relative specific growth rate (A), relative carbohydrate content (B) and flocculation intensity(C) of the mutants and the wild strain of *S. platensis* after the 10^th^ subculture.

After the ARTP mutagenesis for *S. platensis*, 62 mutants of *S. platensis* were obtained from the 96-well microplate after 10 days culture. These 62 mutants were transferred to 48-well plates for subsequent subcultures of 10 days. The specific growth rate of the mutants and wild strain at different subcultures were listed in Supporting Information (Figure S1). As to the first subculture, we got a 72.6% positive mutation frequency based on the growth rate. During the subsequent subculture, some mutants, such as 4-A5, 4-B8, 4-B10 and 4-C7, were presumably unstable and recovered, causing the change in the mutant distribution with the subculture. While some mutants, such as 1-3, 3-B9, 3-A10 and 4-C11，showed stability with high growth rate. Figure 5 showed the change in mutation frequency in terms of specific growth rate with the subcultures, indicating that the mutation frequency was trended to be stable after 9^th^ subculture, with 45% total mutation frequency and 25% positive mutation frequency in terms of specific growth rate (Figure 5). After 10^th^ subculture, the specific growth rate (Figure 6A), carbohydrate content and the flocculation intensity of the mutants were evaluated and compared with the wild strain in 50 ml flask culture of 10 days. In the mutant library, 32.3% of the mutants showed increase in carbohydrate content by over 20% compared with wild type (Figure 6B), and 12.9% of the mutants exhibited the increase in flocculation intensity by more than 100% compared with wild type (Figure 6C).

### 3: Analysis of the representative mutants

**Figure 7:**
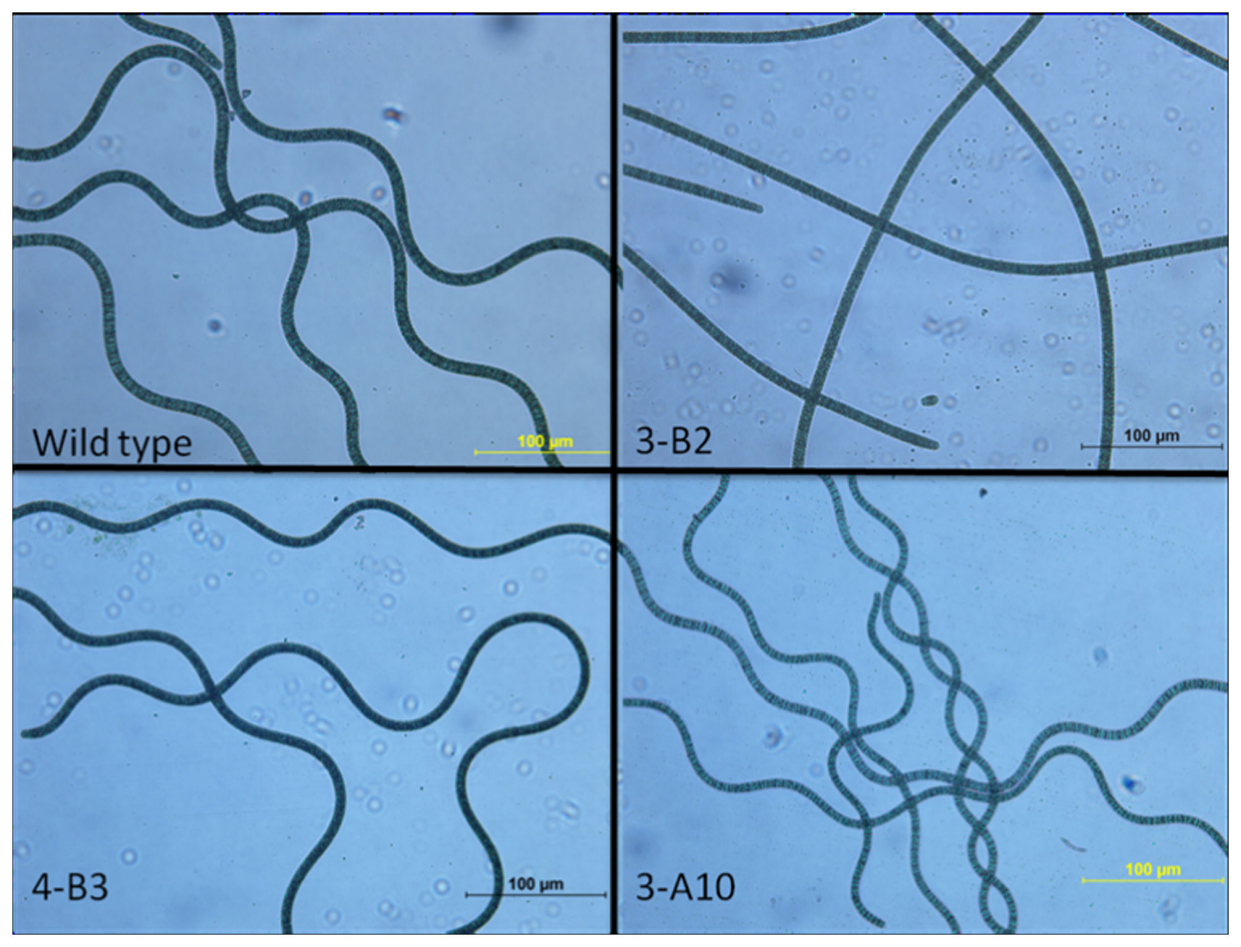
Comparison of morphology of the representative mutants and the wild strain of *S. platensis*.

From the mutant library after 10^th^ subculture, 3 representative mutants were selected to analyze their characteristics in 200 mL flask culture of two weeks: 3-A10, with the slower initial growth rate but higher carbohydrate content and no flocculation intensity; 3-B2, with higher carbohydrate content, higher growth rate and improved flocculation intensity; and 4-B3, with higher growth rate, carbohydrate content similar to wild strain and higher flocculation intensity. Table 1 listed the characteristics of these three mutants and comparison with the wild strain after 10^th^ subculture. The mutant of 4-B3 showed the highest growth rate of 0.093 g/(L×d), 3-B2 showed flocculation intensity of 92.5%, and carbohydrate content of 33.1%, which was increased by 78.0% than the wild type. Moreover, if the carbohydrate content was assumed to be constant during the culture, from the data of specific growth rate and the carbohydrate content, the carbohydrate productivity could be estimated. Mutant of 3-B2 also showed the highest carbohydrate productivity of 26.0 mg/(L×d) which was 65.6% higher than the wild type (Table 1). It was also found that chlorophyll content was higher in the mutants with improved growth rate (Table 1). Figure 7 showed the morphology of the 3 mutants and the wild strain. The mutant of 3-B2 had the big change from the original spiral of the wild strain to linear filament. The stability of carbohydrate content of different mutants and wild strain (control) during different subcultures was shown in Table 2, indicating high genetic stability.

**Table 1:** Characteristics of the mutants and the wild strain (control) (n=3).

	Growth rate (g/L×d)	Flocculation (%)	Carbohydrates content (g/g)	Carbohydrates productivity (mg/L×d )	Chlorophyll (mg/g)
Control	0.0842±0.018	63.6±6.13	0.186±0.045	15.7±3.8	3.53±0.069
3-A10	0.0583±0.016	46.1±3.65	0.261±0.051	15.2±3.0	1.07±0.008
3-B2	0.0786±0.017	92.3±9.33	0.331±0.049	26.0±3.9	3.51±0.040
4-B3	0.0930±0.025	40.7±1.83	0.131±0.046	12.2±4.3	4.52±0.446

**Table 2:** Stability of carbohydrate content (g/g) of different mutants and wild strain (control) during different subcultures (n=3).

subculture	11	15	20
Control	0.177±0.022	0.184±0.012	0.186±0.011
3-A10	0.265±0.021	0.249±0.032	0.261±0.025
3-B2	0.333±0.027	0.327±0.024	0.331±0.014
4-B3	0.134±0.020	0.141±0.026	0.131±0.031

## Discussion

In this study, we successfully applied a novel ARTP mutagenesis tool for the first time to mutate *S. platensis* spirals for construction of a mutant library with altered carbohydrate content, growth rate and flocculation intensity. Since *S. platensis* is a multicellular microorganism, single cell might be more feasible for ARTP mutagenesis. Actually, we tried to make single cells from *S. platensis* spirals and we succeeded in making single cells by sonication. However, it was quite difficult for the separated single cells to survive, and after overnight recovering culture, only few cells survived, making them impossible to be mutated by ARTP treatment. Therefore, in this study, *S. platensis* spirals were used directly for ARTP mutagenesis. The ARTP mutagenesis has been succeeded in mutation breeding of many kinds microbes [32,35,41-43]. As shown in this study, the long spirals of *S. platensis* were cut into shorter ones, indicating the breakage effect of ARTP on *S. platensis*, which proved the direct interaction of the plasma and *S. platensis*. The ARTP driven by pure helium contains a large amount of activated chemical species[32,33,36,44,45], which can penetrate the cell wall and cell membrane, and then damage DNA and proteins, thereby creating mutation via damaging the genome in the cells [33]. At the determined RF power input and *D* (the distance between the plasma torch nozzle exit and the sample plate), ARTP radiation time is proportional to the plasma dosage, i.e., mutagenesis dosage. When the ARTP treatment period was more than 60 seconds, the cells of *S. platensis* were all dead (data not shown). The possible reasons might be that, after the long period of ARTP treatment, single cells from the spirals of *S. platensis* were released, presumably leading to the difficulty in recovery, and/or apart from the plasma action, when the spirals were exposed to the helium gas flow for long time, the water of the medium would be brought away to make cells dried, causing the death of *S. platensis*.

For construction of *S. platensis* mutant library, a high throughput screening method for the mutants is of importance. The gliding growth properties and slow growth of *S. platensis* on the solid medium cause the difficulty in using the colony formation for mutant screening from the ARTP-treated spirals[24]. In this study, we successfully established a dilution method using 96-well microplates combined with the microscope observation to put at most one filament into each well of the microplates and to screen the mutants with microplate reader after the ARTP mutagenesis of multicellular *S. platensis* (Figure 2). For the multicellular *S. platensis*, sonication to generate the fragmented filaments of *S. platensis* prior to mutagenesis is reportedly helpful to improve mutagenesis [24]. After ARTP treatment, the filaments of *S. platensis* were significantly cut into short fragments (Figure 3), presumably contributing to the high mutation frequency. However, the short spirals were still multicellular, which might contain different DNA damage degree and different DNA damage locations for the different cells in a spiral and/or different spirals, probably causing the mutants with different phenotypes. Therefore, controlling the number of the shortly cut filaments in one well of microplates to minimum value by dilution was effective to screening of the mutants after ARTP treatment on *S. platensis*. Moreover, during the subculture of 10 days of the mutants using 48-well microoplates for screening, some of them would be enriched. The percentage of mutants with higher or lower growth rate changed as the subculture times increased, so the characteristics of the mutants showed difference between different subcultures before reaching the stable state (Figure S1 in Supporting Material and Figure 5). In fact, after more than the 9^th^ subculture, the specific growth rates of the mutants became stable (Figure 5). 

In addition, some mutants showed the morphology changes. Previous studies showed that the pitch and diameter of the spirals had significant relation with the photosynthesis activity and biomass growth [24,46]. Among the mutant library, morphology of the mutant 3-B2 was changed from spiral to near linear shape. Wang and Zhao reported that the morphogenesis of *S. platensis* filaments from spiral to linear shape was due to DNA mutation [47]. Wu et al. found that UV broke the spiral structure and suggested that, over long-time radiation, adaptation of *S. platensis* to solar UVR could bring about changes in the spiral structure from rather loose helix to a very compressed helix[48]. In this study, the mutant 3-B2 changed its shape just after 3~4 subcultures after the ARTP mutation, and the change of spirals from spiral shape to linear shape might be related to the highest flocculation intensity of the mutant 3-B2 (Table 1). This result also suggested that, ARTP treatment caused the genome mutation of *S. platensis*.

In this research, we selected 3 representative mutants with different phenotypes, 3-A10, 3-B2 and 4-B3. The high genetic stability in terms of carbohydrate content of these representative mutants was confirmed by subcultures (Table 2). Compared with the wild strain, the mutant of 3-B2 with similar growth rate, higher flocculation intensity, higher carbohydrate content (33.1%) and higher carbohydrate productivity (26.0 mg/L×d ), which was 65.6% higher than the wild type, would increase the productivity of *S. platensis* biomass as the fermentation feedstock for the further application. The different phenotypes among the 3 mutants also demonstrated the efficiency of ARTP mutagenesis method implying that ARTP mutagenesis could also be used to generate the mutants of *S. platensis* with tolerance to CO_2_ and salt or other factors , which is undergoing now. Moreover, the undergoing genome sequencing and comparative analysis of these 3 mutants and wild strain showed that the metabolic networks of carbon fixation and metabolites were changed after mutagenesis (data not shown), and this study will elucidate the genome mutation mechanism by ARTP mutagenesis, which are responsible for these different phenotypes, thus helpful to guide the further strain modification to create ideal mutants. 

## Supporting Information

Figure S1 **Comparison of relative specific growth rates of the mutants generated by ARTP mutagenesis and wild strain of *S. platensis* after different subculture.** Relative specific growth rates of 1^st^(A) ,2^nd^(B) ,5^th^(C) and 9^th^(D) subculture.
(DOCX)
