# Supplementary figures and images for: Rapid Mutation of Spirulina platensis by a New Mutagenesis System of Atmospheric and Room Temperature Plasmas (ARTP) and Generation of a Mutant Library with Diverse Phenotypes
Source: PLoS One. 2013 Oct 11;8(10):e77046. doi: 10.1371/journal.pone.0077046 (PMC3851916; doi:10.1371/journal.pone.0077046)

Supporting Information


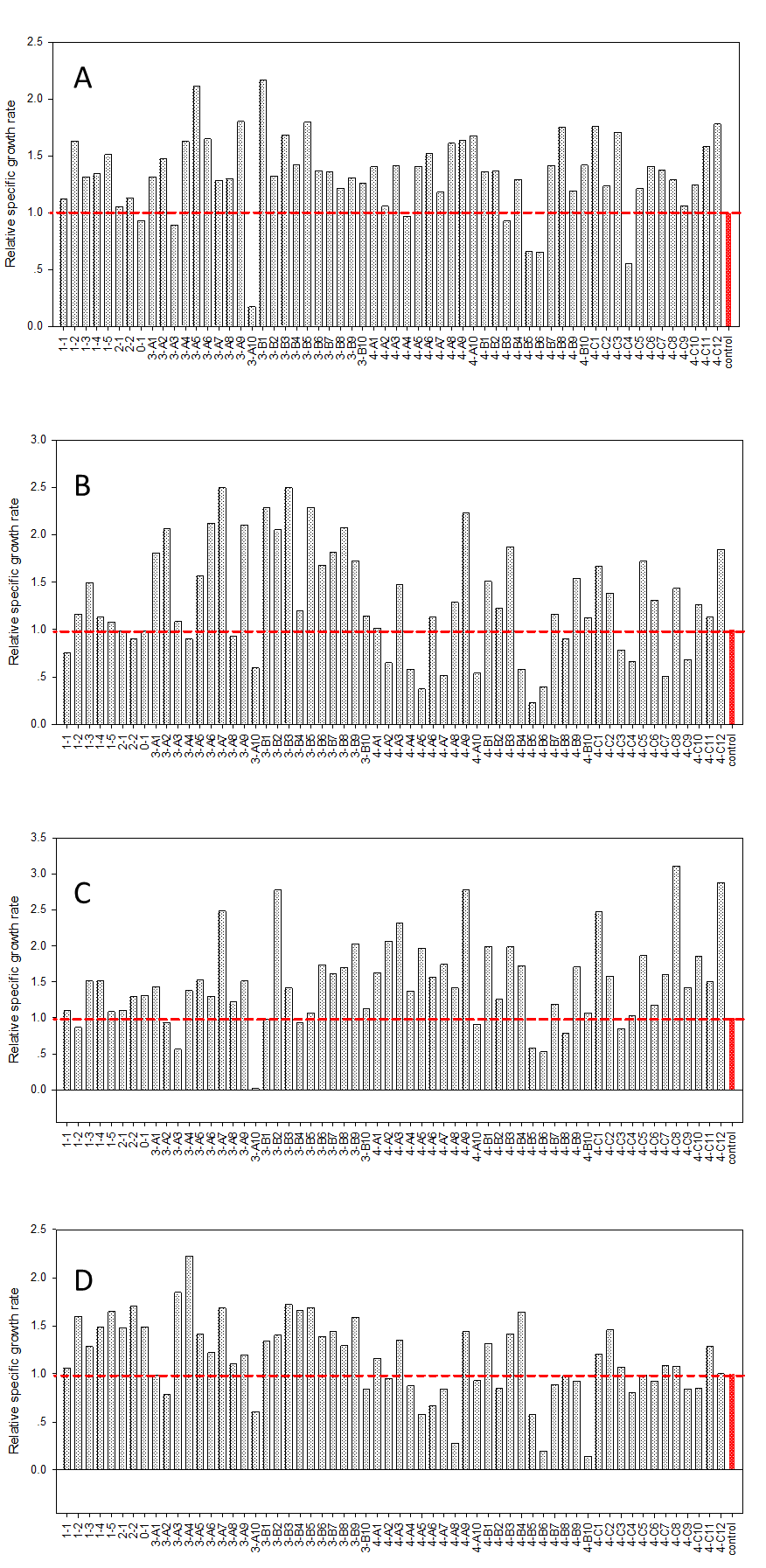

Supplement: Figure S1 — Comparison of relative specific growth rates of the mutants generated by ARTP mutagenesis and wild strain of S. platensis after different subculture. Relative specific growth rates of 1st(A) ,2nd(B) ,5th(C) and 9th(D) subculture. (DOCX) [file pone.0077046.s001.docx]
